# Using Biosensor Devices and Ecological Momentary Assessment to Measure Emotion Regulation Processes: Pilot Observational Study With Dialectical Behavior Therapy

**DOI:** 10.2196/60035

**Published:** 2024-10-09

**Authors:** Shireen L Rizvi, Allison K Ruork, Qingqing Yin, April Yeager, Madison E Taylor, Evan M Kleiman

**Affiliations:** 1 Graduate School of Applied and Professional Psychology, Rutgers University, 152 Frelinghuysen Road, Piscataway, NJ, 08854, United States, 1 8484453914; 2 Evidence-Based Practice Institute, Seattle, WA, United States; 3 Department of Psychology, Rutgers University, Piscataway, NJ, United States; 4 Department of Psychology, University of California Irvine, Irvine, CA, United States

**Keywords:** wearable device, ecological momentary assessment, emotion regulation, psychotherapy mechanisms, dialectical behavior therapy, wearable, wristwatch, novel technology, psychological, treatment, pilot study, adult, personality disorder, mental health, mobile phone, EMA, observational study

## Abstract

**Background:**

Novel technologies, such as ecological momentary assessment (EMA) and wearable biosensor wristwatches, are increasingly being used to assess outcomes and mechanisms of change in psychological treatments. However, there is still a dearth of information on the feasibility and acceptability of these technologies and whether they can be reliably used to measure variables of interest.

**Objective:**

Our objectives were to assess the feasibility and acceptability of incorporating these technologies into dialectical behavior therapy and conduct a pilot evaluation of whether these technologies can be used to assess emotion regulation processes and associated problems over the course of treatment.

**Methods:**

A total of 20 adults with borderline personality disorder were enrolled in a 6-month course of dialectical behavior therapy. For 1 week out of every treatment month, participants were asked to complete EMA 6 times a day and to wear a biosensor watch. Each EMA assessment included measures of several negative affect and suicidal thinking, among other items. We used multilevel correlations to assess the contemporaneous association between electrodermal activity and 11 negative emotional states reported via EMA. A multilevel regression was conducted in which changes in composite ratings of suicidal thinking were regressed onto changes in negative affect.

**Results:**

On average, participants completed 54.39% (SD 33.1%) of all EMA (range 4.7%‐92.4%). They also wore the device for an average of 9.52 (SD 6.47) hours per day and for 92.6% of all days. Importantly, no associations were found between emotional state and electrodermal activity, whether examining a composite of all high-arousal negative emotions or individual emotional states (within-person *r* ranged from −0.026 to −0.109). Smaller changes in negative affect composite scores were associated with greater suicidal thinking ratings at the subsequent timepoint, beyond the effect of suicidal thinking at the initial timepoint.

**Conclusions:**

Results indicated moderate overall compliance with EMA and wearing the watch; however, there was no concurrence between EMA and wristwatch data on emotions. This pilot study raises questions about the reliability and validity of these technologies incorporated into treatment studies to evaluate emotion regulation mechanisms.

## Introduction

There is growing research interest in evaluating proposed mechanisms of change within evidence-based psychological interventions. This work is necessary to understand how and when treatments are effective and to improve and refine these interventions. With such research comes increased reliance on novel technologies to assess variables of interest. These technologies include ecological momentary assessment (EMA) and wearable biosensor wristwatches, which are increasingly used to assess outcomes and mechanisms of change in psychological interventions [1,2]. There is also interest in using these technologies to measure dynamic and mechanistic psychotherapy processes, which could replace assessment approaches that have relied on static and infrequent measures [3].

There are several reasons for the enthusiasm for EMA and wearable sensors in clinical research. The use of these more objective measures (in the case of wearable sensors) could reduce subjective bias in reporting. Using both EMA with multiple prompts per day and wearable sensors would lead to data being obtained in the individual’s natural environment (as opposed to therapy sessions) which would aid in the understanding of contextual factors and symptom variability that can be assessed at the person level [1,4]. Finally, some research has indicated that EMA is more sensitive to change than self-report measures of depression and anxiety [5,6]. Together, these reasons make a strong rationale for incorporating these technologies into treatment research to assess treatment effects at a more granular level. However, despite these reasons for using technologies to address questions regarding mechanisms and processes of treatment, there has been limited attention paid to the overall feasibility and acceptability of using such methods. Such research is critically important because if studies find that compliance rates are low or the technologies are not otherwise feasible to incorporate into treatment research, the benefits of their use are limited.

Two recent studies with nonclinical samples have looked at adherence to intensive data collection methods (EMA, smartwatches, and chest patches) [7,8]. In a study of 45 healthy adults, King et al [8] found generally high compliance rates to EMA prompts (78.92%) and no indication that compliance dropped over time. However, the study lasted only 10 days so implications for longer-term studies are unclear. Ponnada et al [7] report on the preliminary results of a study with healthy young adults in which participants are asked to complete 4-day EMA bursts every 2 weeks. This study used “microinteraction EMA” which restricted the EMA items to simple questions answered directly on the smartwatch with a single tap. Results from a subsample of participants who had completed at least six months of the study (n=81) indicated a compliance rate of 67% to EMA prompts. This is a promising compliance rate for a long-term study; however, the use of healthy young adults and single-item questions may not translate to clinical samples in treatment mechanism studies.

In terms of research with clinical samples, very few studies have incorporated experience sampling methodology into treatment studies. In a study of 55 adults with depression, Eddington et al [9] used a program that automatically called participants 8 times a day for 1 week at baseline and 1 week posttreatment. During those calls, participants were asked 31 questions using prerecorded voice prompts that they answered by using their phone keypad. Among the treatment completers (n=29), compliance rates significantly dropped from 72.23% of call completion during the baseline week to 63.66% of calls during the posttreatment week. In a study of a 1-week internet-based intervention for individuals with social anxiety, Daniel et al [10] had participants complete 5 weeks of EMA (7 surveys a day). Similarly, they found EMA compliance to drop as the EMA period went on, suggesting that the participant burden was high and reduced the incentive to complete. Both of these studies have implications for incorporating technologies into longer-term treatment models.

Another important point for the incorporation of novel methodologies is that it is challenging for research on feasibility and acceptability to keep pace with technological advances. Often, by the time research is published, the technology that was used is outdated which also has implications for the generalizability of findings. For example, an early study [11] incorporating EMA into outpatient psychotherapy used a standalone iPod for participants to carry. EMA compliance rates were not provided, making it difficult to determine how acceptable this technology was for the participants. Furthermore, there remains a lack of consensus in the field regarding design standards and how to capture variables of interest while incorporating such technology into treatment [12]. For example, although interest in capturing emotional processes via EMA is high, there currently exists no standard for operationalizing emotion regulation using EMA [13]. In this study, we operationalized emotion regulation as the presence of high negative affect followed by a subsequent reduction.

In terms of wearable biosensor devices, there is a relatively large body of laboratory-based literature that has examined electrodermal activity (EDA; how conductive is electricity to one’s skin, also called skin conductance level) as an index of physiological arousal that corresponds to the experience of high arousal emotions [14,15]. However, there has been far less exploration of the extent to which ambulatory EDA measures correspond to momentary reports of emotion. If EDA and momentary reports of negative emotion do not correspond, then there is likely limited use in using EDA to detect the changes in emotion associated with emotion regulation. The aims of this study were (1) to evaluate the feasibility and acceptability of incorporating EMA and wearable devices into standard dialectical behavior therapy (DBT) and (2) to conduct an exploratory analysis to evaluate whether these technologies can be used to assess emotion regulation processes and their relation to suicide thinking.

## Methods

### Ethical Considerations

This study was reviewed and approved by the Rutgers University institutional review board (Pro2019001864). All participants provided informed consent as part of the recruitment process and agreed for their deidentified data to be used for research purposes. Participants were compensated US $50‐60 in gift cards for each assessment, excluding the baseline assessment for which there was no compensation.

### Participants

Participants were 20 individuals with borderline personality disorder (BPD) who underwent 6 months of standard DBT in a university-based training clinic. The overall clinic procedures are registered (see ClinicalTrials.org, NCT03123198); however, this substudy of 20 sequentially admitted participants is not. Inclusion criteria required that study participants (1) be 18 years of age or older, (2) agree to take part in research assessments and video recording of therapy and assessments, (3) agree to pay sliding scale fee for treatment sessions, (4) maintain residence within commuting distance of the clinic (<45 minutes), (5) agree to discontinue other forms of therapy (excluding psychotropic medication management), (6) meet diagnostic criteria for BPD, and (7) have an iOS or Android smartphone compatible with our EMA software (MetricWire; ie, from the past 5 years). Potential participants were excluded if they (1) required mental health services not covered by DBT (eg, schizophrenia and life-threatening anorexia nervosa); (2) were non–English-speaking; (3) presented indication of intellectual disability; or (4) were unable to understand consent forms. All participants provided written informed consent for inclusion in the study.

### Measures

Of relevance to this study are variables collected via EMA and wearable devices.

#### Ecological Momentary Assessment

At each EMA prompt, we assessed ratings of negative affect, suicide ideation, as well as variables unrelated to the study (eg, urges to engage in other harmful behaviors like drug use). Participants were asked to rate on a scale from 0 to 5 how much they felt each of the emotions of anxious, sad, agitated, irritated, shame, guilt, self-hate, angry, hopeless, lonely, and burdensome. We created an overall negative affect composite that combined these 11 variables for each prompt (within-person reliability α=.89 and between-person α=.95) and a high-arousal negative affect composite that combined agitated, angry, anxious, and irritated (within-person reliability α=.78 and between-person α=.95). Additionally, at each prompt, participants were asked to answer 2 questions on a 0‐5 scale—“Right now, how strong is your desire to kill yourself” and “Right now, how strong is your desire to stay alive” (reverse coded). These 2 items were combined to create a suicide ideation (SI) composite score (within-person reliability α=.53 and between-person α=.67).

#### Wearable Device Assessment

The first 11 participants were provided the Empatica Embrace and the last 9 participants the Phillips Healthband. Both devices included an accelerometer that can be used to derive information on movement and sleep. The Embrace measured EDA at 4 Hz with electrodes facing the top of the first. The Healthband measured blood volume pressure using a photoplethysmograph at 32 Hz in order to derive heart rate (HR). Both EDA and HR can be used as markers of physiological arousal possibly associated with high arousal negative emotion (and the regulation of this emotion). However, raw HR data are not available for the Healthband, meaning that we could not use these data to explore physiological correlates of emotion regulation. Thus, we focus only on EDA from the Embrace for this study.

### Procedure

#### Recruitment, Screening, and Assessment

Participants self-referred to the clinic and were briefly screened via telephone to determine initial eligibility. Interested and eligible clients were scheduled for an assessment to provide informed consent, confirm eligibility, and complete diagnostic interviews and self-report measures. These meetings were conducted by a graduate student or postdoctoral fellow in clinical psychology, under the supervision of the first author. Given the timing of the study within the COVID-19 pandemic, 85% (n=17) of the intake assessments were conducted via Health Insurance Portability and Accountability Act (HIPAA)–compliant Zoom (Zoom Video Communications). If the client was eligible for the study and interested in participation, they were oriented to the EMA and wearable device procedures. Participants then began DBT, completing standard assessments again at midtreatment, posttreatment, and 3-month follow-up.

#### EMA and Wearable Device

Participants were asked to complete EMA prompts and wear the wristwatch for 1 week of every treatment month, yielding up to 6 weeks of data for each participant. For EMA, participants were prompted 6 times per day, with the first and last prompts based on user-identified sleep and wake times and the remainder sent randomly within prespecified windows. A total of 5 of these surveys were shorter assessments of momentary effect and related factors. The final survey was a longer nightly survey, which contained the items in the random survey plus other items reflecting on the day. Participants were compensated US $0.25 for each of the 5 daily momentary surveys completed and US $0.50 for completing each nightly survey. Participants received an additional US $1.00 per day each for completing 4 or more surveys and wearing the physiological monitor for 6 or more consecutive hours each day. As a bonus, participants were compensated US $5.00 for each week they wore the biosensor for at least 5 days (for 6 consecutive hours each day).

#### DBT Treatment

Treatment providers were clinical psychology graduate students or postdoctoral fellows who were supervised by the first author and completed fundamental coursework in DBT. Clients completed 6 months of comprehensive DBT including weekly individual therapy, weekly group skills training, and intersession skills coaching per the treatment manuals [16,17]. Clients who missed 4 consecutive individual therapy appointments or group skills training sessions were considered treatment dropouts. Fees for services were assigned on a sliding scale determined by household income ranging from US $10 to US $100 per week. Due to the onset of the COVID-19 pandemic, the vast majority of the treatment was delivered via telehealth.

### Data Analysis

To assess feasibility, descriptive statistics were used to summarize compliance rates with EMA and the wearable device over time. To determine whether these technologies can be used to assess emotion regulation processes, we conceptualized the process as what happens within the same day after a high negative affect instance. Thus, we examined all EMA data where we had 2 consecutive data points within 24 hours of each other and where the first data point (T1) was >0.5 SD above the participant mean on a negative affect composite variable. To explore how psychophysiological data corresponded to emotion, we focused on the EDA data that were collected with the Embrace watch. To examine the correspondence between EDA and emotion, we first removed all the data that were likely recorded when the device was not being worn; specifically, we removed any data where the device detected temperature that was unlikely to be skin temperature (ie, <30 °C). We then preprocessed the EDA data using the *signal* R package (R Core Team) [18] by (1) upsampling it to 8 Hz and (2) applying a Butterworth filter to “flatten” the signal (ie, reduce possible noise). After preprocessing, we averaged all EDA data that occurred in the 60 seconds before each EMA prompt. This pre-EMA measure of EDA was used to examine correlations with each affected state, as well as an overall negative affect composite (all 11 emotions) and a high-arousal negative affect composite (agitated, angry, anxious, and irritated). We calculated in the *psych* R package [19] the average between-person correlations (ie, the average of each person’s mean EDA and mean EMA) and within-person (ie, the average of each person’s within-person correlation matrix across momentary observations) as our index of correspondence. Given the high number of correlations being examined here, we also provide a Holm-corrected *P* value that adjusts for potential type I error by using a “step-down” approach where the lowest *P* value is divided by the total number of analyses, the next lowest is divided by the number of analyses minus 1, and so on. Finally, we were interested in examining whether there were changes in suicidal ideation during instances of high negative affect and subsequent change. To evaluate these relationships, a multilevel regression was conducted using the *lme4* package in R [20], where changes in a composite rating of SI were regressed onto changes in negative affect composite score while controlling time in treatment.

## Results

### Participant Characteristics

Participants’ average age was 28.45 (SD 10.93; range 19-65) years. A total of 16 participants (80%) identified as female. Participants identified their race as Caucasian (n=16, 80%), Asian (n=3, 15%), or Black or African American (n=1, 5%), and 20% (n=4) identified as Hispanic. Most participants reported some college (n=9, 45%) or college (n=3, 15%) as their highest level of education, with others reporting completing at least some graduate school (n=5, 25%) and high school (n=3, 15%). Most participants (n=17, 85%) reported being prescribed psychotropic medications. On average, participants reported 2.85 (SD 1.81) current and 3.55 (SD 2.35) lifetime comorbid psychiatric disorders as measured by the Structured Clinical Interview for DSM-5 Disorders (SCID-5) [21]. In terms of treatment compliance, participants attended an average of 24.55 (SD 5.38; range 8‐36) individual therapy sessions and 20.85 (SD 4.49; range 9‐25) group skills training sessions. One (5%) participant dropped out after 2 months of treatment.

### Compliance With Technologies

**Figure 1.**
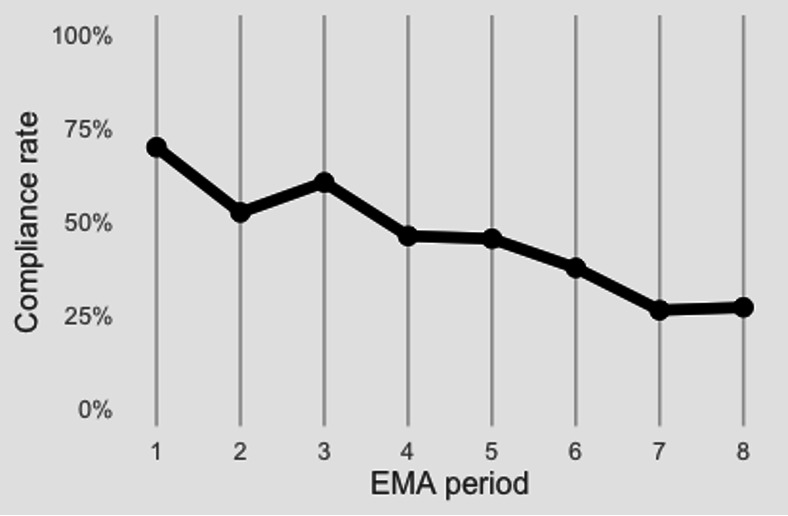
EMA compliance changes over the course of study involvement. EMA: ecological momentary assessment.

On average, participants completed 54.39% (SD 33.1%) of all EMA surveys. Compliance ranged from 4.7% to 92.4%. Participants responded to at least 1 survey on 76.33% of days, and 2 or more on 58.3% of days. EMA compliance decreased over the course of the study; average compliance was >50% for the first 4 data collection weeks and then dropped below 50% in the fifth week of data collection (see Figure 1).

Participants wore 1 of the 2 biosensors—Empatica Embrace and Philips Healthband. Participants (n=11) wore the Embrace on average for 9.52 hours per day during the weeks they were asked to wear the device (SD 6.47 hours) and wore the device for at least some amount of time on 92.6% of all days. Participants (n=9) wore the Healthband for 10.58 hours per day during the weeks they were asked to wear the device (SD 10.51 hours) and wore the device for at least some of the time on 74.6% of days.

### Assessing Emotion Regulation Processes Via EMA

For these analyses, an opportunity for emotion regulation processes to occur was operationalized to be (1) a higher rating (>0.5 SD above the participant mean) on an overall negative affect composite variable that (2) had a subsequent data point within 24 hours (*k*=230; n=12). A total of 8 participants were not included in the analyses due to a lack of observations that fit these parameters. There was a high degree of variability in the negative affect composite over the course of the study at both the within- and between-person level (intraclass correlation=0.51). In addition, there was a decrease in negative affect (ie, change >0.5 SD) from T1 to T2 in 55.35% (*k*=128) of these emotion regulation opportunities, an increase in 35.36% (*k*=82) of the opportunities, and a change within ±0.5 SD in 8.29% (*k*=20) of the opportunities. Table 1 shows the results of the correlations between EMA and EDA. As can be seen from the table, none of the correlations were significant after the Holm correction. Table 2 shows the relationship between emotion regulation processes and suicide ideation. Smaller changes in negative affect composite scores were associated with greater SI ratings at time 2, beyond the effect of SI ratings at time 1.

**Table 1.** Correlations between EMA^a^ and EDA^b^ data.

Variable	Within person			Between person		
	*r*	*P *value	Holm *p*	*r*	*P* value	Holm *p*
**Composites**						
Negative affect	−0.090	.05	0.514	−0.324	.36	1.00
High arousal negative affect^c^	−0.082	.07	0.668	−0.284	.43	1.00
**Individual states**						
Agitated	−0.057	.22	0.876	−0.406	.25	1.00
Angry	−0.074	.11	0.760	−0.435	.21	1.00
Anxious	−0.058	.21	1.00	−0.114	.75	0.753
Burden	−0.044	.34	1.00	−0.296	.41	1.00
Guilt	−0.026	.57	1.00	−0.459	.18	1.00
Hopeless	−0.019	.68	0.683	−0.353	.32	1.00
Lonely	−0.075	.10	0.817	−0.115	.75	1.00
Sad	−0.097	.03	0.379	−0.347	.33	1.00
Self-hate	−0.109	.02	0.207	−0.261	.47	1.00
Shame	−0.060	.19	1.00	−0.344	.33	1.00

a EMA: ecological momentary assessment.

b EDA: electrodermal activity.

c High arousal composite includes agitated, angry, anxious, and irritated.

**Table 2.** Results of regression analysis.

Predictors	Dependent variable: suicidal thinking at T2		
	β (95% CI)		*P* value
**Intercept**	0.599 (0.302 to 0.896)		<.001
**Suicidal thinking at T1**	0.636 (0.572 to 0.701)		<.001
**Change in negative affect from T1 to T2**	−0.097 (−0.107 to 0.086)		<.001
**Random effects**			
σ^2^	1.21		—^a^
τ_00 ID_	0.18		—
Marginal *R*^2^/conditional *R*^2^	0.568/0.625		—

a Not applicable.

## Discussion

The main findings of this study were that compliance with technologies was moderate and dropped over the course of the study and that no associations were found between data obtained from EMA and data obtained via the wearable device. To our knowledge, this pilot study was the first to incorporate both biosensor wearable devices and EMA to measure a key target mechanism and emotion regulation in real time during a psychological intervention (ie, DBT). Although a small sample, there were a number of important findings and their implications lead to concern for future research in this domain.

In terms of feasibility and acceptability, we found a moderate rate of EMA compliance (54%) with at least 1 survey completed on 76.33% of days. This compliance rate is lower than rates found in other EMA studies conducted [22]. This may be related to the long duration of the study, especially since compliance dropped over the course of 6 months. To effectively study emotion regulation in real time and as a function of treatment, it is preferred to have several completed EMA prompts within the same day and for the completed EMA prompts to be relatively stable over time. Our finding that 2 or more prompts were completed on just 58.3% of days indicates that future studies will need to place greater emphasis on increasing the number of surveys completed per day (to examine T1-T2 changes) within clinical samples. Indeed, 8 of the 20 (40%) participants had to be excluded from EMA analyses of emotion regulation processes because they did not provide data that met our measurement parameters. Similarly, compliance with the wearable biosensor device was generally high but waned over time. Our finding that compliance with both technologies waned over the course of the study renders observing treatment effects more difficult. Determining how to keep compliance up over time is likely crucial for evaluating treatment changes.

Our findings that EMA and EDA data were not correlated are noteworthy. Because EMA requires the participant to report their own experiences, it is often considered more “subjective” while psychophysiological data are often seen as more “objective.” However, it is important to recognize that the field does not have a ground truth (gold standard) that states which form of measurement is “accurate.” This lack of consensus coupled with our results makes it difficult to determine which technology needs to improve to increase validity. It is also possible that this lack of correspondence provides counterevidence for the classical view of emotions which suggests emotions have natural and physical essences that may be better captured using perceiver-independent tools (eg, autonomic nervous system activation). Rather, emotions could be multidimensional and different assessment approaches offer unique information that does not necessarily correlate [23].

There are some limitations to the study. First, the sample had only 20 participants and data collection occurred primarily during the pandemic. It is unclear how representative these individuals are of clients in DBT generally. Second, we focused on a few variables of interest in our EMA surveys. Other variables may have proven more reliable or consistent with EDA. The lack of consensus on how to define emotion regulation via EMA leaves researchers to determine appropriate variables themselves. Third, our sample included only individuals diagnosed with BPD. Although this is the target sample for DBT, this population may not represent other therapy populations and studies with different samples may yield different compliance rates. Fourth, because raw HR data were not available, we were unable to include it in these more granular analyses, this is unfortunate given the evidence linking BPD and emotional ability with HR variability [24]. While not without problems (eg, poor data quality for diverse skin) [25], future studies would benefit from including HR.

Although new technologies are often quickly embraced and used in psychological research, our study suggests that researchers should be cautious about using these technologies to measure emotion regulation processes in real time. It is likely that solutions to the problem require effort in engineering (eg, making the devices easier to use) and psychosocial (eg, designing protocols to maximize wear time) domains, as well as advances in broader emotion research. Until these solutions are identified and implemented, continuing to use these technologies, such as EDA, in psychotherapy studies may prove premature and unlikely to yield accurate pictures of treatment mechanisms and processes.

## Abbreviations

BPD: borderline personality disorder
DBT: dialectical behavior therapy
EDA: electrodermal activity
EMA: ecological momentary assessment
HIPAA: Health Insurance Portability and Accountability Act
HR: heart rate
SCID-5: Structured Clinical Interview for DSM-5 Disorders
SI: suicide ideation
